# The Impact of Timing of Pertussis Vaccination During Pregnancy on Infant Antibody Levels at Birth: A Multi-Country Analysis

**DOI:** 10.3389/fimmu.2022.913922

**Published:** 2022-06-28

**Authors:** Justin Gomme, Nasamon Wanlapakorn, Hoang Thi Thu Ha, Elke Leuridan, Sereina Annik Herzog, Kirsten Maertens

**Affiliations:** ^1^ Centre for the Evaluation of Vaccination, Vaccine and Infectious Diseases Institute, University of Antwerp, Antwerp, Belgium; ^2^ Department of Pediatrics, Center of Excellence in Clinical Virology, Bangkok, Thailand; ^3^ Division of Academic Affairs, Faculty of Medicine, Chulalongkorn University, Bangkok, Thailand; ^4^ National Institute of Hygiene and Epidemiology, Hanoi, Vietnam; ^5^ Institute for Medical Informatics, Statistics and Documentation, Medical University of Graz, Graz, Austria; ^6^ Centre for Health Economics Research and Modelling Infectious Diseases, Vaccine and Infectious Diseases Institute, University of Antwerp, Antwerp, Belgium

**Keywords:** Tdap, vaccination, pregnancy, timing, multi-country analysis

## Abstract

**Background:**

Pertussis vaccination during pregnancy is an effective strategy at reducing pertussis-related morbidity and mortality in infancy and is recommended across several countries. However, the optimal timepoint for vaccination in pregnancy to afford maximal protection to newborns is yet to be elucidated. This multi-country analysis aimed to model the impact of timing of vaccination during pregnancy on infant antibody titers at birth.

**Methods:**

A multi-country analysis on a cohort of mother-infant pairs (n=698) vaccinated between 19.6-37.1 weeks gestation was conducted. Data taken from four parent studies on pertussis vaccination during pregnancy were modelled using natural cubic splines and linear mixed models to study the association of both gestational age at vaccination and the interval between vaccination and delivery with pertussis-specific cord blood antibody levels after pertussis vaccination during pregnancy.

**Results:**

Term born infants on average achieve the highest antibody levels at birth if women are vaccinated before 31 weeks’ gestation. When considering both term and preterm deliveries, an interval of at least 7.5 weeks between vaccination and delivery is required to achieve the highest cord blood antibody levels. The models show that vaccinating earlier than these timeframes will also provide the infant with equally high antibody levels at birth.

**Conclusions:**

Vaccinating in the second and early third trimester results in the highest antibody levels at birth. Vaccinating earlier within this window is needed to provide equal benefits to both term and preterm born infants.

## Background

Despite the availability of successful universal pertussis immunization programs, the World Health Organization (WHO) reported in 2014 an estimated 24.1 million pertussis cases and 160,700 pertussis related deaths in children below 5 years of age (1, 2). As most national immunization schedules only recommend pertussis containing vaccines to be administered from 6-8 weeks of age, infants below 2 months of age remain unprotected against pertussis. Therefore, the highest incidence, hospitalization and mortality rates are observed in this population (3, 4).

Vaccination with a tetanus, diphtheria, acellular Pertussis (aP) (Tdap) vaccine during pregnancy is being implemented in an increasing number of countries to combat neonatal pertussis-related morbidity and mortality. Administering a Tdap vaccine during pregnancy prompts an immune response in women resulting in an increase in pertussis-specific antibody levels which are transferred to the foetus through the placenta. This yields improved infant immunity in the first months of life and offers protection against pertussis disease (5, 6).

Although Tdap vaccination during pregnancy is implemented in many countries, national health services tend to recommend their own optimal window to vaccinate during pregnancy. There is no consensus in national recommendations as the optimal timing to vaccinate during pregnancy to convey maximum immunity to the infant (7–10). Most countries recommend vaccination in the late second or third trimester of pregnancy. However, more recently several countries have recommended vaccinations earlier in pregnancy to reach as many pregnant women as possible with the UK recommending as early as 16 weeks gestational age. Vaccination after 36 weeks has been found less effective as although antibody levels reach a maximum 2 weeks after vaccination, additional time is required for these antibodies to pass to the infant (11).

This multi-country analysis investigates the effect of timing of Tdap vaccination during pregnancy on pertussis-specific infant antibody levels at birth in both term and preterm born infants to define an optimal timeframe for vaccination in pregnancy to achieve high antibody titers at birth in all neonates. The first aim (Aim 1) of the study was to determine the effect of gestational age at vaccination (GAV) on infant pertussis antibody titers at birth in term born infants.

Secondly (Aim 2), we determined the effect of the interval between vaccination and delivery on infant pertussis-specific antibody titers at birth in both term and preterm born infants.

## Methods

### Study Population

This multi-country analysis utilises data from four parent studies conducted in either Belgium, Vietnam or Thailand (9, 12–15). All parent studies were prospective cohort studies looking at the effect of Tdap vaccination during pregnancy on maternal and infant immune responses. The study population of the four parent studies was divided into five study groups for this analysis according to the differing protocols of the original study (**Table 1**).

**Table 1:** Overview of the study groups (BE1: Belgium 1; VN1: Vietnam 1; TL1: Thailand 1; BE2: Belgium 2; BE3: Belgium 3).

Study group	Parent study	Country	Number of mothers	Number of infants	Term/preterm born	Maternal vaccine^‡^
BE1	MATAB 4^(14)^	Belgium	57	59	term	Boostrix^®^
VN1	MATAB 4^(13)^	Vietnam	55	55	term	Adacel^®^
TL1	MATAB 5^(9,12)^	Thailand	370	370	term*	Boostrix^®^
BE2	MATAB 6^(15)^	Belgium	112	114	term	Boostrix^®^
BE3	MATAB 6^(15)^	Belgium	82	100	preterm	Boostrix^®^

Infants were classified as term or preterm born for analysis on an individual basis, i.e. <37 weeks GAD as preterm and ≥37 weeks GAD as term born. *Not all infants were carried to term in the study groups classified as term; a small proportion of women still had GAD <37 weeks (but ≥36 weeks GAD). ‡Maternal vaccines: Boostrix^®^ (GSK Biologicals, Rixensart, Belgium) composition: 5 Lf of Tetanus Toxoid (TT), 2.5 Lf of Diphtheria Toxoid (DT), 8 µg of inactivated PT, 8 µg of FHA and 2.5 µg of PRN; Adacel^®^ (Sanofi Pasteur, Canada) composition: 5 Lf of TT, 2 Lf of DT, 2.5 µg of inactivated PT, 5 µg of FHA, 3 µg of PRN and 5 µg of fimbriae types 2 and 3.

Pertussis-specific antibody concentrations (Pertussis Toxin (PT), Filamentous Hemagglutinin (FHA), Pertactin (PRN)) in blood taken from infants at birth (cord) were used in this analysis. Full details on recruitment, data collection, inclusion/exclusion criteria and laboratory testing can be found in the articles published on the parent studies (9, 12–15). Written informed consent was obtained from all participants in the parent studies. Women and infants were excluded from this multi-country analysis if they were missing measurements for specific antibody titers, or had missing values for just GAV (Aim 1: effect of GAV), or either GAV or gestational age at delivery (GAD) (Aim 2: effect of interval between GAV and GAD).

### Statistical Analysis

Descriptive statistics are reported as means with standard deviation (SD) or medians with range or interquartile range (IQR). Laboratory results are reported as geometric mean concentrations (GMCs) with 95% confidence intervals (CI) and are expressed in IU/mL (BE1, VN1, TL1) or EU/mL (BE2, BE3). Antibody titers below the detection limit were replaced by half the lower limit of quantification (16).

Linear Mixed Models (LMMs) were used to analyse the effect of GAV (Aim 1) and interval between GAV and GAD (Aim 2), respectively, on pertussis antibody titers at birth (details below). The relationship between GAV and antibody titers at birth is investigated only in term born infants. Due to the lower GAD, a preterm born infant from a woman vaccinated during pregnancy has less time for transplacental antibody transfer compared to a term born infant. As such, we considered GAV not as a reliable predictor when looking at recommendations intended to include both term and preterm deliveries. Therefore, we used the interval between GAV and GAD (in weeks) as a predictor when investigating term and preterm born infants. One observation from MATAB 6 with GAV 13.4 weeks was removed from analysis as there were no further observations in the vicinity to lend validity to associated findings.

Information on covariates was collected upon enrolment or at each study visit; depending on parent study. Covariates of interest related to the mother were age at delivery, country of residence, parity, maternal vaccine, and number of days delivered before/after the due date. Infant covariates were sex, birth weight in grams, whether the infant was part of a twin, and information on preterm/term delivery if needed. Preterm infants were defined as deliveries at <37 weeks’ gestation.

LMMs were constructed using the *lmer* function (R-package: *lme4*) (17). Natural cubic splines with predefined boundary knots at 5^th^ and 95^th^ percentiles of the data were used to model the non-linear nature of the association (R-package: *splines*) (18). All models included a random intercept to account for clustering within study groups. Models were constructed by backwards stepwise selection. Selection of variables and degrees of freedom of cubic splines were based on Akaike Information Criterion (AIC) values (19). Interaction terms were investigated using the *anova* function. Continuous variables were assessed for collinearity. Covariates exhibiting a moderate correlation or greater were not included in the same model (20). Results of the LMMs are presented as graphical representations, keeping stated variables constant to visualise the effect of timing of vaccination during pregnancy on cord antibody titers. CIs were calculated using the *bootMer* (R-package: *lme4*) (17). Plots were constructed using the ggplot function (R-package: *ggplot2*) (21).

Missing outcome and key exposure measures were treated as missing completely at random (MCAR) and complete case analyses were conducted. Due to the likelihood-based methods of LMMs, missing covariate data were treated as missing at random (MAR) (22). The amount of missing covariate data was limited (max: 2.0%). Sensitivity analyses on the robustness of the models to the presence of twins in the dataset were carried out by comparing parameter estimates and standard error for models to alternative models including twins in different ways as described by Bruyndonck (23).

P-values < 0.05 and changes in AIC ≥ 2 were considered as statistically significant. All analyses were performed using statistical software R (version 3.6.3) (18).

## Results

### Demographic Characteristics

Demographic characteristics of the complete parent population and the study population by aim are given in **Table 2**.

**Table 2:** General characteristics of parent population and study population by aim.

	Parent population (all women and infants)	Study population used to determine the effect of Gestational age at Vaccination on cord blood antibody titers in term born infants (Aim 1)	Study population used to determine the effect of interval between Gestational Age at Vaccination and Gestational age at delivery on cord blood antibody titers in term and preterm born infants (Aim 2)
Number of participants	698	475	541
Median Gestational Age at Vaccination (min-max)	30.0 (13.4-37.1)	30.1 (19.6-36.9)	29.9 (19.6-36.9)
Median Gestational Age at Delivery (min-max)	38.9 (28.4-42.4)	39.3 (37.0-42.4)	39.0* (28.4-42.4)
Median Interval Between Vaccination and Delivery in Weeks (min-max)	8.6 (0.4-26.1)	NA	9.0 (0.7-20.4)
Mean Maternal Age at Delivery (SD)	29.9 (5.03)	29.8 (5.25)	29.9 (5.07)
Study Group: n (%)			
BE1	59 (8.5)	56 (11.8)	58 (10.7)
VN1	55 (7.9)	45 (9.5)	50 (9.2)
TL1	370 (53.0)	278 (58.5)	284 (52.5)
BE2	114 (16.3)	96 (20.2)	96 (17.7)
BE3	100 (14.3)	0 (0)	53 (9.8)
Study Country: n (%)			
Belgium	273 (38.9)	152 (32)	208 (38.4)
Vietnam	55 (7.9)	45 (9.5)	49 (9.1)
Thailand	370 (53.2)	278 (58.5)	284 (52.5)
Parity: n (%)			
Primiparous	416 (59.9)	273 (57.5)	314 (58.0)
Multiparous	278 (40.1)	202 (42.5)	227 (42.0)
Preterm-born Infants: n (%)			
Term-born	565 (80.9)	NA	474 (87.6)
Preterm	133 (19.1)	NA	67 (12.4)
Presence of Infants from a Twin Pregnancy: n (%)			
Single birth	654 (93.7)	470 (98.9)	511 (94.5)
Twin	44 (6.3)	5 (1.1)	30 (5.5)

*Median GAD for preterm deliveries was 35.7 weeks (min-max: 28.4-36.9). GAV = gestational age at vaccination, GAD = gestational age at delivery, max = maximum, min = minimum, NA = not applicable, n = number of observations, SD; standard deviation; BE1, Belgium 1; VN1, Vietnam 1; TL1, Thailand 1; BE2, Belgium 2; BE3, Belgium 3.

### Laboratory Results

**Figure 1:**
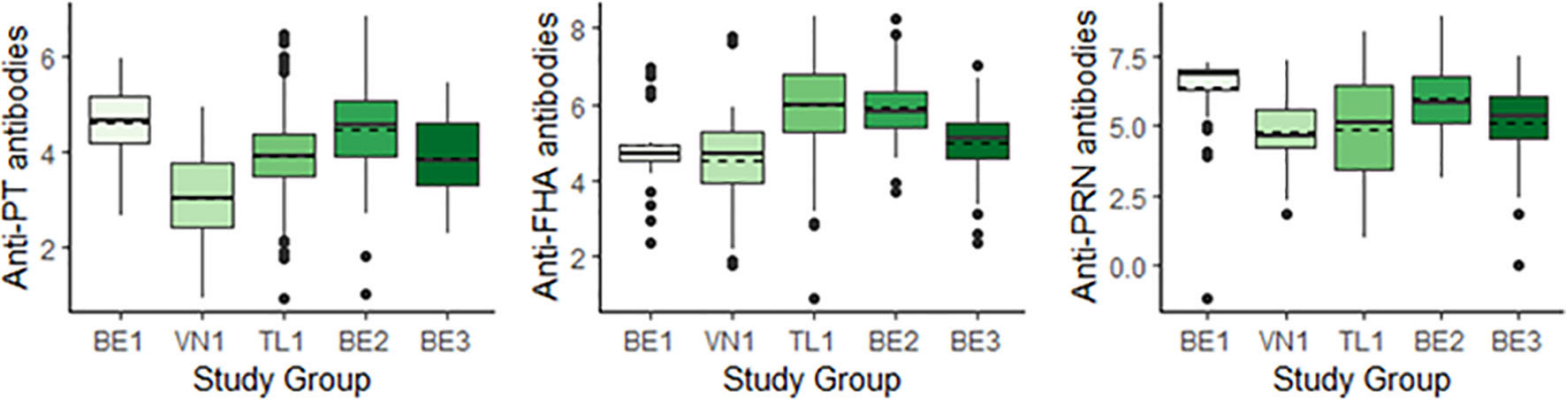
Boxplots comparing cord blood antibody titers across study groups for anti-PT (left), anti-FHA (centre) and anti-PRN antibodies (right), reported in IU/mL (BE1, VN1, TL1) or EU/mL (BE2, BE3). Geometric Mean Concentrations (GMCs) are represented by dashed horizontal lines. BE1, Belgium 1; VN1, Vietnam 1; TL1, Thailand 1; BE2, Belgium 2; BE3, Belgium 3; PT, Pertussis Toxin; FHA, Filamentous Hemagglutinin; PRN, Pertactin.

GMCs within the study populations for both aims are representative of the parent population (**Table 3**). **Figure 1** shows a comparison of GMCs and the distribution of antibody titers measured between the 5 study groups. GMCs were found to differ between individual study groups (**Figure 1**). The highest anti-PT GMCs were measured in BE1 and BE2, and the lowest in VN1. A different trend was observed for anti-FHA antibodies, with BE2 and TL1 recording highest GMCs, and BE1 and VN1 recording lowest. The highest cord anti-PRN antibody GMCs are reported in BE1 and BE2. The other three study groups report similar anti-PRN GMCs.

**Table 3:** GMCs with 95% CIs measured in infants at delivery in total parent population (only 544 infants had information about antibody titers at delivery available) and study groups by aim.

	Anti-PT			Anti-FHA			Anti-PRN		
	Parent Population	Aim 1	Aim 2	Parent Population	Aim 1	Aim 2	Parent Population	Aim 1	Aim 2
n	544	475	541	544	474	541	544	474	541
GMC (95% CI)	3.99 (3.90-4.07)	4.01 (3.91-4.10)	3.99 (3.90-4.08)	5.61 (5.51-5.71)	5.70 (5.59-5.80)	5.61 (5.51-5.71)	5.26 (5.10-5.41)	5.28 (5.11-5.44)	5.26 (5.11-5.41)

*GMCs are expressed in IU/mL (BE1, VN1, TL1) or EU/mL (BE2, BE3), n = number of observations. PT, Pertussis Toxin; FHA, Filamentous Hemagglutinin; PRN, Pertactin. Aim 1: determine the effect of GAV on cord blood antibody titers in term born infants; Aim 2: determine the effect of the interval between GAV and GAD on cord blood antibody titers in term and preterm born infants). GMCs are expressed in IU/mL (BE1, VN1, TL1) or EU/mL (BE2, BE3), n = number of observations.

### Effect of Gestational Age at Vaccination on Cord Blood Antibody Titer

In total, 475 anti-PT, and 474 anti-FHA and anti-PRN cord blood antibody titer measurements were available in term born infants (**Table 3**).

The dependence of cord blood antibody titers to PT, FHA and PRN on GAV in term born infants was modelled by means of natural cubic splines with fixed boundary knots. Different covariates were found significant in models for the different antigens. A brief overview of LMMs constructed are given in **Table 4**.

**Table 4:** Overview of models. *Aim 1: effect of GAV on pertussis-specific antibody titers at birth; Aim 2: effect of interval between GAV and GAD on pertussis-specific antibody titers at birth.

Model	Aim*	Short description	Spline degrees of freedom	Covariates included in model
1	1	LMM showing the association of *GAV* with *cord anti-PT antibodies*	4	Country, birth weight and number of days delivered before/after the due date
2	2	LMM showing the association of *Int* with *cord anti-PT antibodies*	4	Country and birth weight
3	1	LMM showing the association of *GAV* with *cord anti-FHA antibodies*	5	Vaccine type and birth weight
4	2	LMM showing the association of *Int* with *cord anti-FHA antibodies*	3	Birth weight
5	1	LMM showing the association of *GAV* with *cord anti-PRN antibodies*	3	Country, birth weight, and age of mother at birth
6	2	LMM showing the association of *Int* with *cord anti-PRN antibodies*	5	Country, preterm/term born, birth weight and age of mother at birth

GAD, gestational age at delivery; GAV, gestational age at vaccination; Int, interval between GAV and GAD; LMM, Linear Mixed Model; PT, Pertussis Toxin; FHA, Filamentous Hemagglutinin; PRN, Pertactin. *Aim 1: effect of GAV on pertussis-specific antibody titers at birth; Aim 2: effect ofinterval between GAV and GAD on pertussis-specific antibody titers at birth.

**Figure 2:**
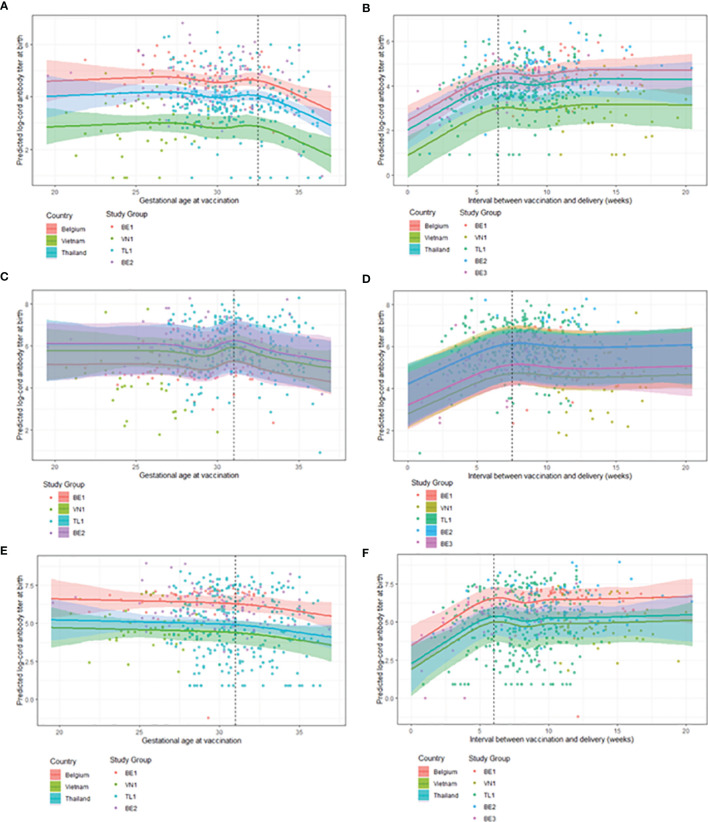
Observed GMCs (points) and model estimates (solid line) with 95% confidence intervals (shaded area) for antibody titers across covariates. Note, results of the LMMs are presented as graphical representations, keeping stated covariates constant to visualise the effect of timing of maternal vaccination on cord antibody titers. **(A)** Model 1: GMC estimates for cord anti-PT titers at delivery by GAV across country with covariates: weight = mean birth weight, BA-EDD = 0 days. **(B)** Model 2: GMC estimates for cord anti-PT titers at delivery by interval between GAV and GAD across country with covariates: weight = mean birth weight. **(C)** Model 3: GMC estimates for cord anti-FHA titers at delivery by GAV across study groups with covariates: weight = mean birth weight, vaccine = Boostrix^®^. **(D)** Model 4: GMC estimates for cord anti-FHA titers at delivery by interval between GAV and GAD across study groups with covariates: weight = mean birth weight. **(E)** Model 5: GMC estimates for cord anti-PRN titers at delivery by GAV across country with covariates: weight = mean birth weight, age = mean age of mother at birth. **(F)** Model 6: GMC estimates for cord anti-PRN titers at delivery by interval between GAV and GAD across country with covariates: weight = mean birth weight, age = mean age of mother at birth, being term born. BA-EDD = number of days delivered before/after the due date, GAD = gestational age at delivery, GAV = gestational age at vaccination, GMC = geometric mean concentration, BE1 = Belgium 1, VN1 = Vietnam 1, TL1 = Thailand 1, BE2 = Belgium 2, BE3 = Belgium 3, PT = Pertussis Toxin, FHA = Filamentous Haemmaglutinin, PRN = Pertactin, LMMs = linear mixed models.

**Figure 2 (panels A, C, E)**, predicted steady cord blood mean antibody levels for women vaccinated between 19.5-32.5 weeks gestation for anti-PT, between 19.5-31.5 weeks gestation for anti-FHA and between 19.5-31.0 weeks gestation for anti-PRN antibodies. For women vaccinated later than these intervals, a tendency to lower cord blood antibody levels start to be recorded, with a consistent steady decrease in antibody titers for all pertussis-specific antigens observed if vaccinated closer to delivery up until 37 weeks’ gestation (the upper limit of the data).

### Effect of Different Time Intervals Between Vaccination and Delivery on Cord Blood Antibody Titer 

After adding preterm deliveries to the dataset, 541 cord blood antibody titer measurements were available for analysis. Half of all preterm born infants in the parent population have missing blood samples at birth and were excluded.

The dependence of cord blood antibody titers to PT, FHA and PRN on interval between GAV and GAD was modelled by means of natural cubic splines. Covariates included in individual models and degrees of freedom are shown in **Table 4**.

Term and preterm infants born to women vaccinated during pregnancy can expect highest cord blood anti-PT antibody titers if they are vaccinated 6.5-20.5 weeks prior to delivery (**Figure 2B**). The highest cord blood antibody titers against FHA and PRN were found in infants born to women vaccinated 7.5-20.5 and 6.0-20.5 weeks prior to delivery, respectively (**Figures 2D, F**). Mean estimates are stable for cord blood antibody titers between 6.0/7.5-20.5 weeks. When vaccinating closer to delivery than the lowest value of the interval, a steady decrease in antibody titers is seen as the interval between GAV and GAD tends to 0.

### Covariates Influencing the Impact of Gestational Age at Vaccination and Interval Between Vaccination and Delivery on Cord Blood Antibody Titers 

Several factors were found to be influential in the final model estimates for antibody titer (**Table 4**). The covariates varied depending on the antigen investigated but did not impact the cut-off point for GAV or the interval between GAV and GAD beyond which lower antibody titers are observed, e.g. lower anti-PT titers were always estimated when vaccinating after 32.5 weeks GA, regardless of infant weight. In Models 1, 2, 5 and 6, country was found influential and explains a large amount of variability in the data. In Models 3 and 4, country was not included and the variable study group explains the largest amount of variability in the data.

In Models 3 and 4, the use of different maternal vaccines also has an impact on cord blood antibody titers. In Model 3, where the variable vaccine was included, study group BE1 (women receiving Boostrix^®^) has the lowest predicted GMCs. However, in Model 4 where the variable vaccine was not included, VN1 (women receiving Adacel^®^) have the lowest predicted GMCs.

## Discussion

Although some studies have already demonstrated the benefits of vaccinating earlier in pregnancy to achieve high antibody titers in infants at birth, the optimal timeframe for Tdap vaccination during pregnancy is still under debate (7–10). This analysis investigates the impact of timing of Tdap vaccination in pregnancy on infant antibody titers at birth using data from different studies conducted in several countries, applying a modelling approach over a continuous range of GAV spanning from 19.5-37.0 weeks. Our results show clear association between timing of vaccination during pregnancy and cord blood antibody titers. In our models, the highest infant antibody titers at delivery are achieved in women vaccinated earlier than 31.0-32.5 weeks gestation for term born infants and at least 6.0-7.5 weeks before delivery for both term and preterm born infants, with variations observed across the different pertussis-specific antigens. Based on our models, women vaccinated towards the end of current recommended national guidelines will not be affording their infants the highest possible antibody titers at birth, especially if the infant is born preterm.

When considering the effect of GAV on cord blood antibody titers in term born infants, our models show the highest titers against PT, FHA and PRN in women vaccinated in the second, and early third trimester of pregnancy before 32.5, 31.5 and 31 weeks gestation respectively. A drop in cord blood antibody titers is observed with increasing GAV in the third trimester of pregnancy. This corresponds with what has already been reported in literature where several studies report higher antibody titers in cord blood from infants born to women vaccinated early in the third trimester than those vaccinated later in pregnancy (10, 24, 25).

The study by Eberhardt et al. even suggested that higher antibody titers in cord blood are achieved in infants born to women vaccinated between 13.0-25.0 weeks gestation compared to those vaccinated after 25 weeks (8). However, our analysis did not show notable differences in antibody titers across women vaccinated between 19.5-31.0 weeks gestation. A reason for these conflicting results could be that a disproportionate amount of women within Eberhardt’s study group vaccinated after 25 weeks were vaccinated later, rather than earlier in the third trimester. The lower cord blood antibody titers observed in women vaccinated closer to delivery are in accordance with the saturable nature of transplacental IgG antibody transfer and longer *in-utero* exposures to high antibody concentrations (26).

When including preterm born infants in the analysis, the results reinforce the conclusions made on GAV. The models estimate that an interval of at least 6.0-7.5 weeks is required between vaccination and delivery to achieve the highest antibody titers in infants at birth, with cord blood antibody titer estimates plateauing for vaccinations further from delivery. The minimum interval between GAV and GAD to afford the highest antibody titers at birth is therefore estimated to be greater than 7.5 weeks before delivery. This interval corresponds with the optimal GAV found for term born infants, but the results for term born infants do not hold for preterm infants due to their lower GAD. Therefore, vaccination earlier in pregnancy is necessary to protect both term and preterm born infants as we are unable to accurately predict how far into a pregnancy a woman will deliver.

Due to the timing of implementation of infant pertussis vaccination schedules in Vietnam (1981) and Thailand (1977), the vaccine administered during pregnancy in these countries could be the first aP containing vaccine the women are receiving in their life, whereas it is likely a booster vaccination in Belgian women (27, 28). As such, women are likely to experience different immune responses in pregnancy based on whether this was a priming or booster vaccine dose. Consequently, country was shown to be an influential covariate in most models, accounting for large differences in GMC estimates. In models where country was not included, the largest differences in estimated cord blood antibody titers were observed between study groups. Although the same covariates are not present in each model, the information conveyed to the models by these variables is very similar as individual study groups did not span multiple countries.

The vaccines Boostrix^®^ and Adacel^®^ contain differing compositions of antigens and could therefore elicit different immune responses. This relationship is demonstrated in Models 3 and 4. Model 3 (effect of GAV on cord anti-FHA antibodies) estimates infants in VN1 to exhibit higher anti-FHA antibody titers than infants in BE1 if vaccine is kept constant. However, type of vaccine was not included in Model 4 (effect of Int on cord anti-FHA antibodies) and the difference in vaccine compositions is no longer explained by an independent variable. As such, infants in BE1 are estimated to exhibit higher antibody titers than those in VN1 in Model 4.

A key strength of our analysis is the large sample size allowing us to model GAV on a continuous scale. This allowed us to closer pinpoint after which timepoint of vaccination during pregnancy lower antibody titers are expected in the infant at birth. We analyse data spread across different countries, containing the biggest collection of pertussis-specific antibody data for preterm infants to date. This allows us to draw more global conclusions from our results with greater generalisability. Due to the distribution of the data, our models can be deemed to be most reliable between gestational ages 25-35 weeks, and Int <15 weeks, with CIs widening outside these boundaries in all models.

Our analysis had several limitations. Exclusion criteria were not entirely consistent across studies, resulting in baseline differences across study groups. Due to differences in study protocols, certain data were not available in each study, such as data on maternal humoral immune response in pregnancy. Also, this analysis only looked at the impact of timing of vaccination in pregnancy on infant antibody titers at birth, not taking into account the possible impact on T-cell mediated immunity. However, so far, no data are available yet on the transfer of T-cell immunity across the placenta. Another limitation of the analysis is the large proportion of missing blood samples in preterm born infants. Complications are more likely to arise in preterm deliveries; resulting in higher probability of missing blood samples during delivery or withdrawal from the study. As such, we surmise that the data are MAR. Less biased estimates may be possible using multiple imputation. However, as imputation of outcome variables can lead to biases of their own, complete case analysis was deemed to yield valid approximations (29).

To conclude, our results support vaccination in the second and early third trimester of pregnancy in order to maximise protection afforded to newborns through vaccination during pregnancy. In addition, we advocate vaccinating earlier within this window in order to stand a better chance of affording the same benefits to both term and preterm born infants. It is important to take preterm deliveries into account when defining optimal vaccination windows and based on our models many women vaccinated towards the end of national recommendations are not affording their infants the highest possible antibody titers at birth in-pregnancy vaccination and provides additional information to guide policymaking.

## Data Availability Statement

Dataset contains personal information of subjects and is therefore not publicly available. Requests to access these datasets should be directed to kirsten.maertens@uantwerpen.be.

## Ethics Statement

The studies involving human participants were reviewed and approved by the Ethics committee of the University Hospital Antwerp. Written informed consent to participate in this study was provided by the parents of the participant or the participants' legal guardian/next of kin.

## Author Contributions

KM was responsible for the conception of the work. KM, EL, NW, and HTTH executed the data collection. JG, SH, and KM performed the data-analysis and the interpretation. JG was responsible for drafting the article. All authors performed a critical revision of the article and approved the final version for publication.

## Funding

SH acknowledges funding by a grant from the European Research Council (ERC) under the European Union’s Horizon 2020 research and innovation program (grant agreement 682540 TransMID).

## Conflict of Interest

The authors declare that the research was conducted in the absence of any commercial or financial relationships that could be construed as a potential conflict of interest.

## Publisher’s Note

All claims expressed in this article are solely those of the authors and do not necessarily represent those of their affiliated organizations, or those of the publisher, the editors and the reviewers. Any product that may be evaluated in this article, or claim that may be made by its manufacturer, is not guaranteed or endorsed by the publisher.

## References

[1] World Health Organization. Global Health Observatory (GHO) Data: Diphtheria-Tetanus-Pertussis (DTP3) Immunization Coverage. Available at: https://www.who.int/data/gho/data/themes/immunization.

[2] Yeung KHT Duclos P Nelson EAS Hutubessy RCW. An Update of the Global Burden of Pertussis in Children Younger Than 5 Years: A Modelling Study. Lancet Infect Dis (2017) 17(9):974–80. doi: 10.1016/S1473-3099(17)30390-0 28623146

[3] Kapil P Merkel TJ. Pertussis Vaccines and Protective Immunity. Curr Opin Immunol (2019) 59:72–8. doi: 10.1016/j.coi.2019.03.006 PMC6774807 31078081

[4] World Health Organization. WHO Vaccine-Preventable Diseases: Monitoring System. 2020 Global Summary. (2020).

[5] Abu-Raya B Maertens K. Protection of the Newborn Through Vaccination in Pregnancy. Neoreviews (2021) 22(1):e25–39. doi: 10.1542/neo.22-1-e25 33386312

[6] Abu-Raya B Maertens K Edwards KM Omer SB Englund JA Flanagan KL. Global Perspectives on Immunization During Pregnancy and Priorities for Future Research and Development: An International Consensus Statement. Front Immunol (2020) 11:1282. doi: 10.3389/fimmu.2020.01282 32670282 PMC7326941

[7] Vaz-de-Lima LRA Sato HK Fernandes EG Sato APS Pawloski LC Tondella ML. Association Between the Timing of Maternal Vaccination and Newborns' Anti-Pertussis Toxin Antibody Levels. Vaccine (2019) 37(36):5474–80. doi: 10.1016/j.vaccine.2019.04.079 PMC7419216 31153689

[8] Eberhardt CS Blanchard-Rohner G Lemaître B Boukrid M Combescure C Othenin-Girard V. Maternal Immunization Earlier in Pregnancy Maximizes Antibody Transfer and Expected Infant Seropositivity Against Pertussis. Clin Infect Dis (2016) 62(7):829–36. doi: 10.1093/cid/ciw027 PMC4787611 26797213

[9] Wanlapakorn N Maertens K Chaithongwongwatthana S Srimuan D Suratannon N Vongpunsawad S. Assessing the Reactogenicity of Tdap Vaccine Administered During Pregnancy and Antibodies to Bordetella Pertussis Antigens in Maternal and Cord Sera of Thai Women. Vaccine (2018) 36(11):1453–9. doi: 10.1016/j.vaccine.2018.01.059 29426663

[10] Naidu MA Muljadi R Davies-Tuck ML Wallace EM Giles ML. The Optimal Gestation for Pertussis Vaccination During Pregnancy: A Prospective Cohort Study. Am J Obstetrics Gynecology (2016) 215(2):237.e1–.e6. doi: 10.1016/j.ajog.2016.03.002 26968625

[11] Public Health England. Pertussis (Whooping Cough) Vaccination Programme for Pregnant Women. Information for Healthcare Providers (2020). Available at: https://assets.publishing.service.gov.uk/government/uploads/system/uploads/attachment_data/file/897039/Pertussis_vaccination_for_pregnant_women_2020.pdf.

[12] Wanlapakorn N Maertens K Vongpunsawad S Puenpa J Tran TMP Hens N. Quantity and Quality of Antibodies After Acellular Versus Whole-Cell Pertussis Vaccines in Infants Born to Mothers Who Received Tetanus, Diphtheria, and Acellular Pertussis Vaccine During Pregnancy: A Randomized Trial. Clin Infect Dis (2020) 71(1):72–80. doi: 10.1093/cid/ciz778 31418814

[13] Hoang HT Leuridan E Maertens K Nguyen TD Hens N Vu NH. Pertussis Vaccination During Pregnancy in Vietnam: Results of a Randomized Controlled Trial. Vaccine (2016) 34(1):151–9. doi: 10.1016/j.vaccine.2015.10.098 26529073

[14] Maertens K Caboré RN Huygen K Hens N Van Damme P Leuridan E. Pertussis Vaccination During Pregnancy in Belgium: Results of a Prospective Controlled Cohort Study. Vaccine (2016) 34(1):142–50. doi: 10.1016/j.vaccine.2015.10.100 26592142

[15] Maertens K Orije MRP Herzog SA Mahieu LM Hens N Van Damme P. Pertussis Immunization During Pregnancy: Assessment of the Role of Maternal Antibodies on Immune Responses in Term and Preterm-Born Infants. Clin Infect Dis (2022) 74(2):189–98. doi: 10.1093/cid/ciab424 33971009

[16] Tran TMP Abrams S Aerts M Maertens K Hens N. Measuring Association Among Censored Antibody Titer Data. Stat Med (2021) 40(16):3740–61. doi: 10.1002/sim.8995 33942345 PMC8251995

[17] Douglas Bates MM Bolker B Walker S. Fitting Linear Mixed-Effects Models Using Lme4. (2015) J Stat Software 67(1):1–482015. doi: 10.18637/jss.v067.i01

[18] Team RC. R: A Language and Environment for Statistical Computing. Vienna, Austria: R Foundation for Statistical Computing (2020). p. ed2020.

[19] Bozdogan H. Model Selection and Akaike's Information Criterion (AIC): The General Theory and its Analytical Extensions. Psychometrika (1987) 52(3):345–70. doi: 10.1007/BF02294361

[20] Chan YH. Biostatistics 104: Correlational Analysis. Singapore Med J (2003) 44(12):614–9. 14770254

[21] Wickham H. Ggplot2 Package. In: Elegant Graphics for Data Analysis. Springer-Verlag New York; (2016).

[22] West B. T. WKB Galecki AT. Linear Mixed Models: A Practical Guide Using Statistical Software. 2nd ed. Boca Raton, Florida:CRC Press; (2014).

[23] Bruyndonckx R Hens N Aerts M. Simulation-Based Evaluation of the Linear-Mixed Model in the Presence of an Increasing Proportion of Singletons. Biom J (2018) 60(1):49–65. doi: 10.1002/bimj.201700025 29067702

[24] Abu Raya B Srugo I Kessel A Peterman M Bader D Gonen R. The Effect of Timing of Maternal Tetanus, Diphtheria, and Acellular Pertussis (Tdap) Immunization During Pregnancy on Newborn Pertussis Antibody Levels - A Prospective Study. Vaccine (2014) 32(44):5787–93. doi: 10.1016/j.vaccine.2014.08.038 25173476

[25] Healy CM Rench MA Swaim LS Smith EOB Sangi-Haghpeykar H Mathis MH. Association Between Third-Trimester Tdap Immunization and Neonatal Pertussis Antibody Concentration. JAMA (2018) 320(14):1464–70. doi: 10.1001/jama.2018.14298 PMC6233794 30304426

[26] Fouda GG Martinez DR Swamy GK Permar SR. The Impact of IgG Transplacental Transfer on Early Life Immunity. ImmunoHorizons (2018) 2(1):14. doi: 10.4049/immunohorizons.1700057 29457151 PMC5812294

[27] Jit M Dang TTH Friberg I Hoang VM Pham Huy TK Walker N. Thirty Years of Vaccination in Vietnam: Impact and Cost-Effectiveness of the National Expanded Programme on Immunization. Vaccine (2015) 33 Suppl 1(Suppl 1):A233–A9. doi: 10.1016/j.vaccine.2014.12.017 PMC4428532 25919167

[28] Muangchana C Thamapornpilas P Karnkawinpong O. Immunization Policy Development in Thailand: The Role of the Advisory Committee on Immunization Practice. Vaccine (2010) 28:A104–A9. doi: 10.1016/j.vaccine.2010.02.043 20412989

[29] Buuren Sv. Flexible Imputation of Missing Data. 2nd ed. Boca Raton: CRC/Chapman & Hall, FL (2018).

